# Supplementary material for: Switch of cell migration modes orchestrated by changes of three-dimensional lamellipodium structure and intracellular diffusion
Source: Nat Commun. 2023 Aug 24;14:5166. doi: 10.1038/s41467-023-40858-x (PMC10449835; doi:10.1038/s41467-023-40858-x)
Supplement: Supplementary file 1 — Supplementary Information [file 41467_2023_40858_MOESM1_ESM.pdf]

Supplementary Information

Switch of cell migration modes orchestrated by changes of three-dimensional lamellipodium structure and intracellular diffusion

Authors: Chao Jiang, Hong-Yu Luo, Xinpeng Xu, Shuo-Xing Dou, Wei Li, Dongshi Guan, Fangfu Ye, Xiaosong Chen, Ming Guo, Peng-Ye Wang, Hui Li

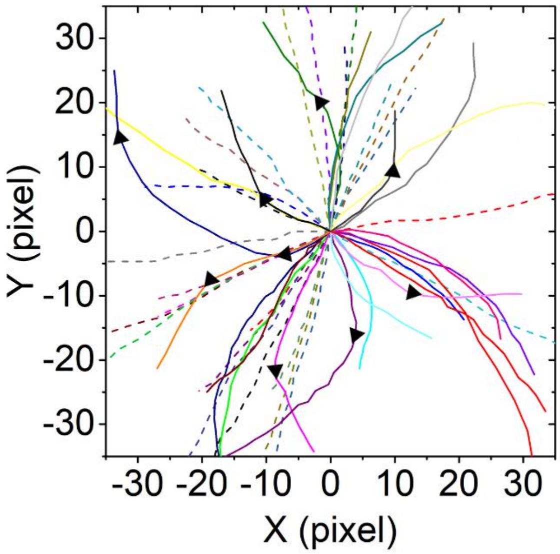

Fig. S1. Keratocyte trajectories in 1 min. The cells usually undergo persistent motion over long distances (dash lines), but occasionally make a short turn (indicated by arrows) and then migrate persistently in a new direction (solid lines). Note that the start points of all trajectories have been shifted to the coordinate origin for display. Trajectory number, 50. Pixel size, 0.2667 μm.

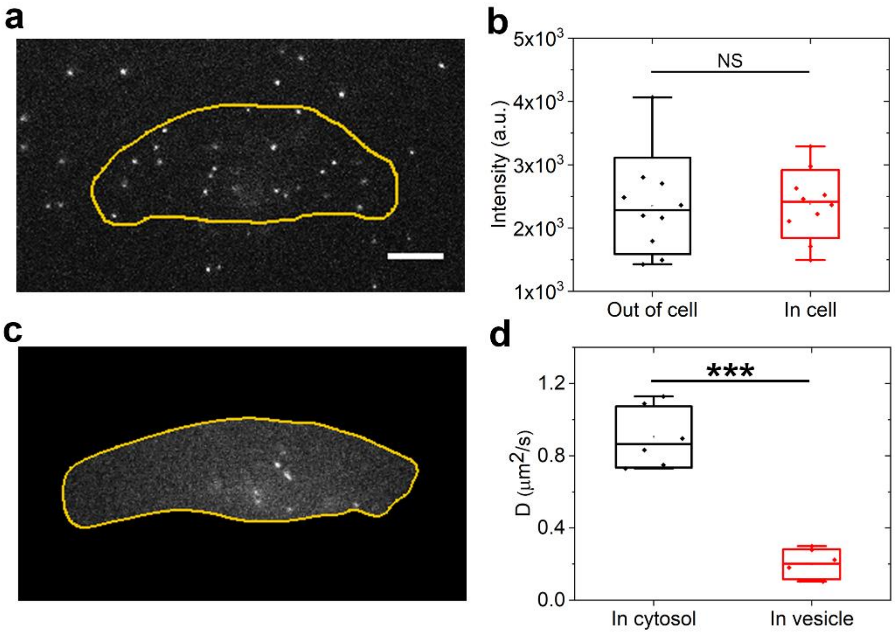

Fig. S2. QDs are dispersed in the cytosol but not trapped in vesicles. a, Fluorescent images of the loaded QDs in the cytosol via the osmotic lysis of pinocytic vesicles, as well as the QDs locating on the cover glass. The cell boundary is marked by the yellow line. **b,** QDs in the cytosol (n = 10) have the same intensities as those individually immobilized on glasses (n = 10), with p = 0.92. **c,** Fluorescent images of endocytic QDs in cells. **d,** The diffusion coefficient of QDs in the cytosol is three times larger than the endocytic QDs trapped in vesicles (n = 6 cells), with p = 3.0E-5. All the boxes represent SD, where the center lines represent the medians, whiskers represent 1.5×outliers and triangles represent the averages. Scale bar, 10 μm.

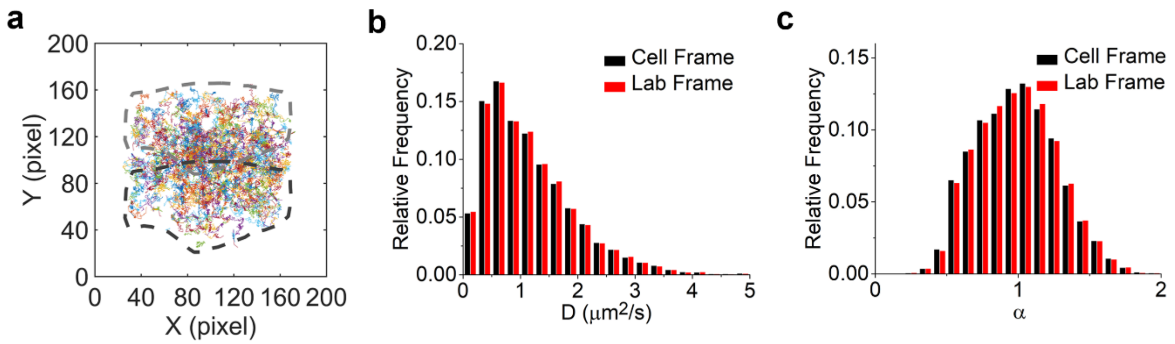

Fig. S3. The cell migration has no obvious influence on the measured QD diffusion. a, In the lab frame, the intracellular QD diffusion trajectories in a moving keratocyte are plotted. The cell migrates upwards with its outlines shown at the start (dark dashed lines) and end (grey dashed lines) time points. **b, c,** Both the measured diffusion rates **(b)** and the exponent α **(c)** of QD diffusion remain the same from the cell frame to the lab frame.

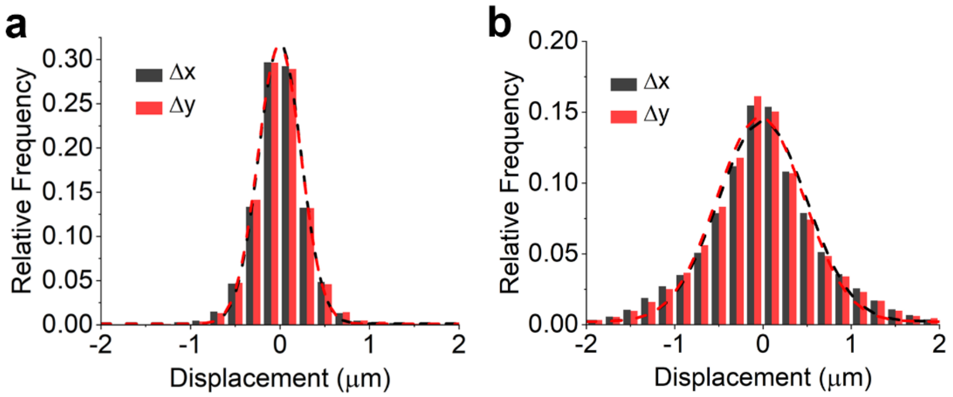

Fig. S4. The intracellular diffusion of QDs is two-dimensional isotropic in migrating cells. Cell migrations are aligned in the *y* direction, with which the intracellular flow is expected to be parallel. The displacements in the *x* and *y* directions for intracellular diffusive QDs are the same both at 30 ms **(a)** and 180 ms **(b)**, indicating that the intracellular flow or actin flow has no detectable influence on the QD diffusion over short time periods in our experiments.

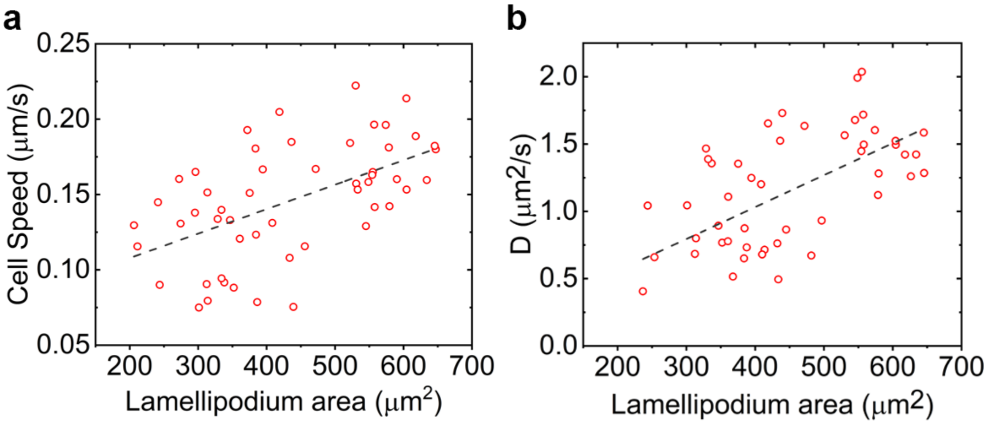

Fig. S5. Positive correlations between lamellipodium area and cell speed (cell, *n* = 52) **(a)**, and between lamellipodium area and intracellular diffusion rate (cell, *n* = 48) **(b)**.

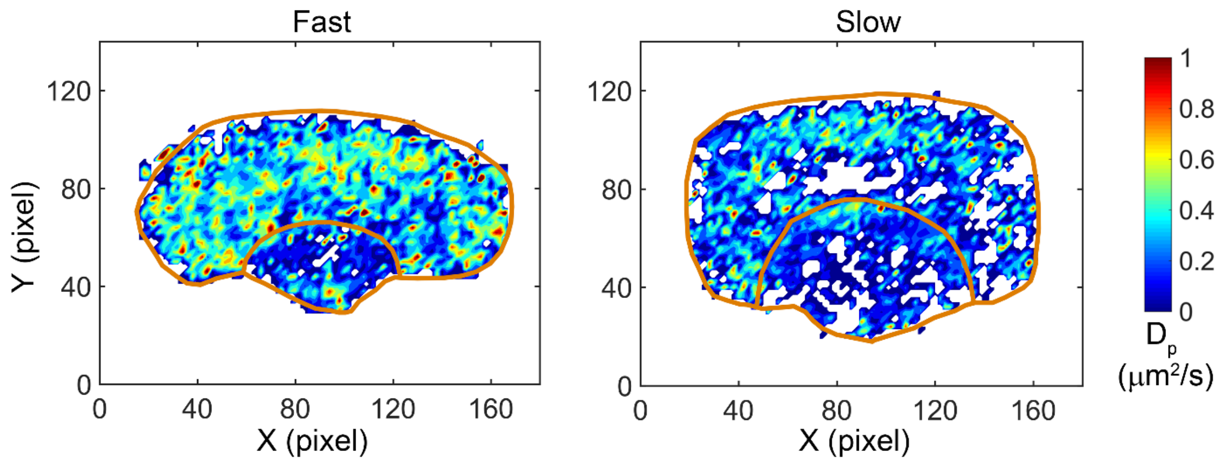

Fig. S6. Diffusion map of tracks aligned from different cells with similar sizes and shapes. The diffusion map of fast cells (left panel, 5 cells, 4231 trajectories) is continuous, whereas that of slow cells (right panel, 5 cells, 2561 trajectories) has decreased diffusion rates with a gap area near the cell body where QDs are excluded.

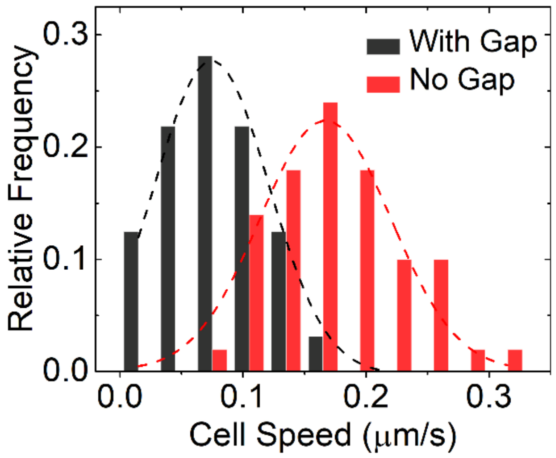

Fig. S7. Cells with a gap (exclusion) region of QD diffusion in lamellipodia are prone to migrate slower than cells without such a gap region. The mean speeds are 0.075 and 0.16 μm/s, respectively. (cells with a gap, n = 35; cells without a gap, n = 50).

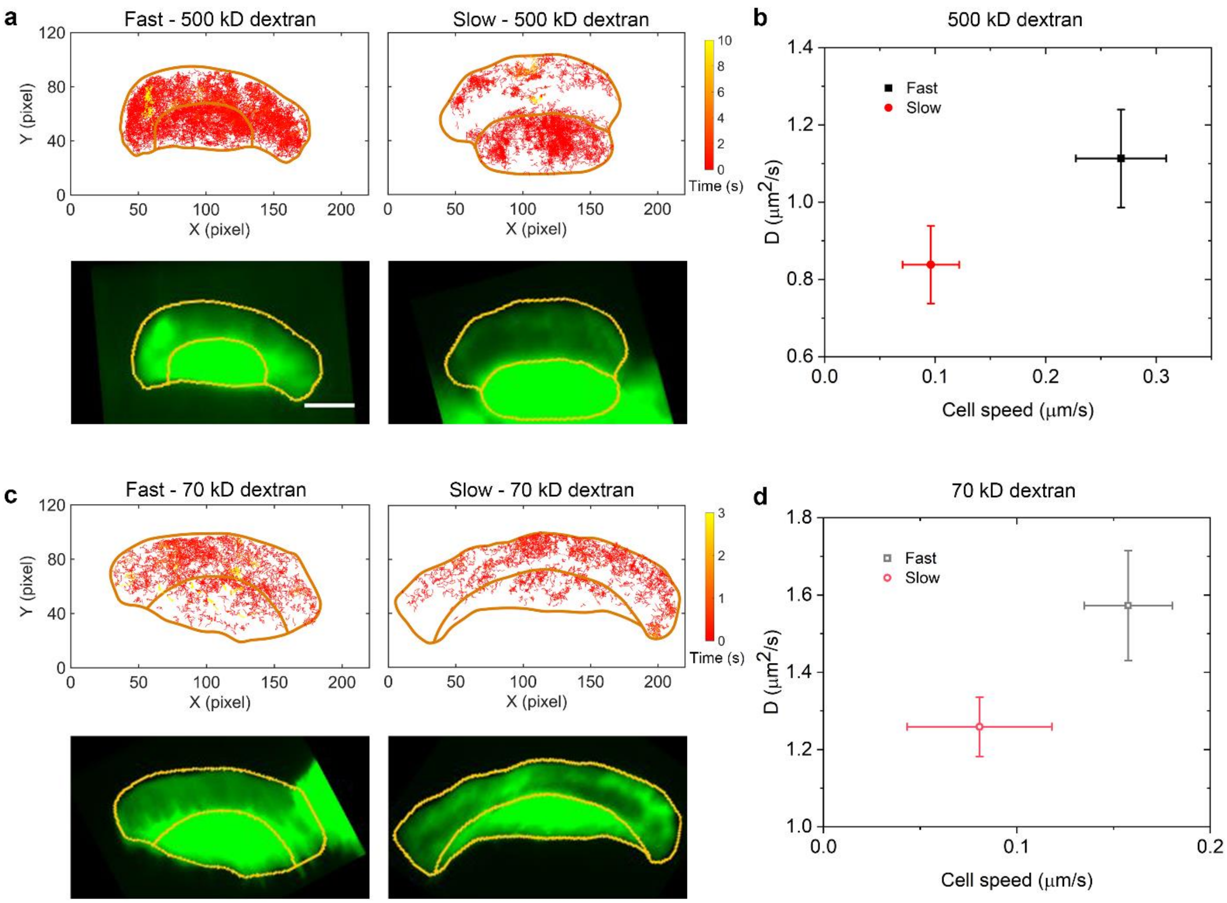

Fig. S8. The diffusion of 500- and 70-kD dextrans in fast and slow migrating keratocytes. a, The trajectories of 500-kD FITC-dextrans (upper panels) and CMRA fluorescent images (lower panels) for migrating cells with fast and slow speeds. **b,** Average diffusion rates of 500-kD FITC-dextrans and migration speeds for cells (fast, n = 5; slow, n = 5). Note that the CMRA (emission 576 nm) is used to indicate the cell volume here, with the color shown in green for consistency. **c,** The trajectories of 70-kD rhodamine B-dextrans (upper panels) and CMFDA fluorescent images (lower panels) for migrating cells with fast and slow speeds. **d,** Average diffusion rates of 70-kD rhodamine B-dextrans and migration speeds for cells (fast, n = 4; slow, n = 4). Scale bar, 10 μm; data are shown as mean±SD.

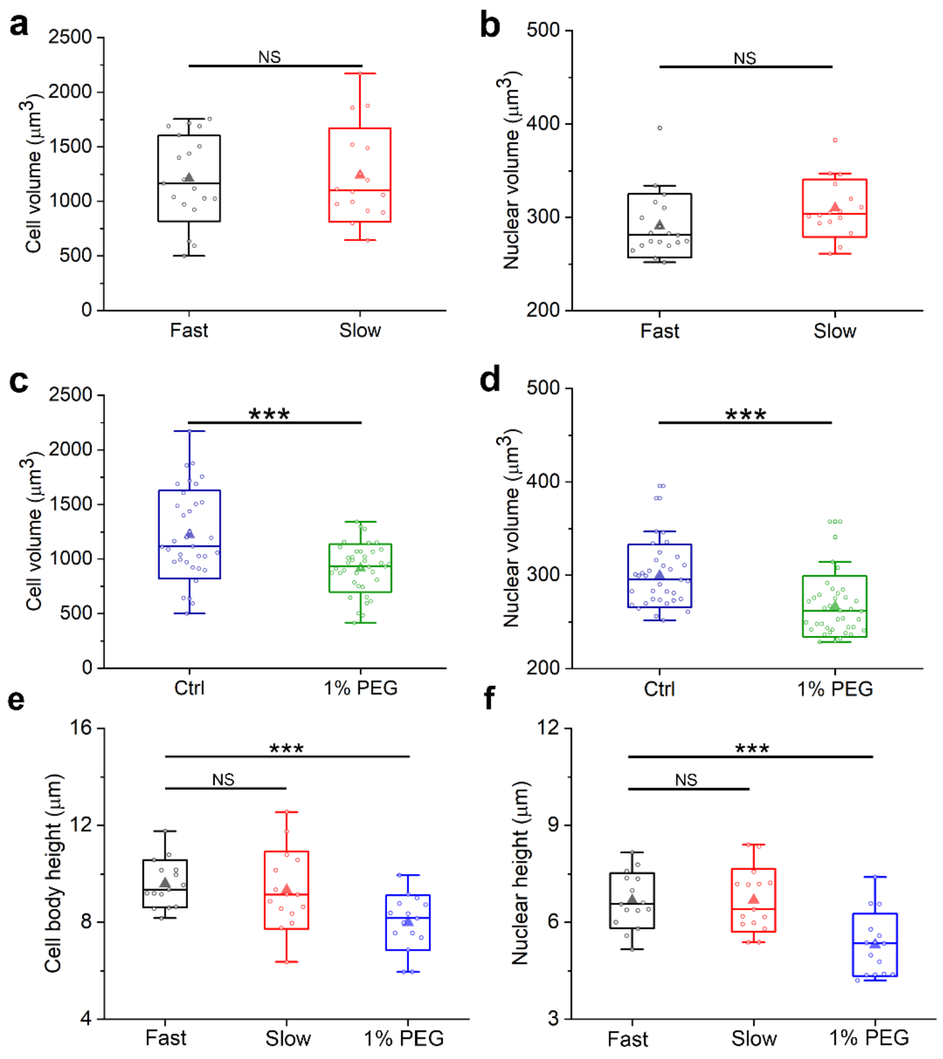

Fig. S9. Characterization of cell and nuclear volumes. a, b, The cell **(a)** and nuclear **(b)** volumes for fast- and slow-moving cells. They remain the same when cells change the migration modes (fast, n = 19; slow, n = 16, p value in **a** is 0.84, p value in **b** is 0.1). **c, d,** Comparison of the cell **(c)** and nuclear **(d)** volumes between control cells and those treated with 1% PEG (ctrl, n = 35; 1% PEG, n = 39), with p = 2.1E-4 in (c) and p = 6.0E-5 in (d). With the 1% PEG treatment, noticeable decreases in both volumes were observed. **e, f,** The heights of cell body **(e)** and nuclei **(f)** for fast- and slow-moving cells, as well as for cells under 1% PEG treatments (n =15). In (e), p = 0.60, fast and slow; p = 2.9E-4, fast and 1% PEG. In (f), p = 0.97, fast and slow; p = 3.0E-4, fast and 1% PEG. All the boxes represent SD, where the center lines represent the medians, whiskers represent 1.5×outliers and triangles represent the averages. The cells were labeled with CMFDA and the nuclei with Hoechst33342. We performed 3D scanning imaging using a confocal laser microscopy. The volumes were measured by counting voxels in the z-stack images, with a 3D pixel size of 200 nm.

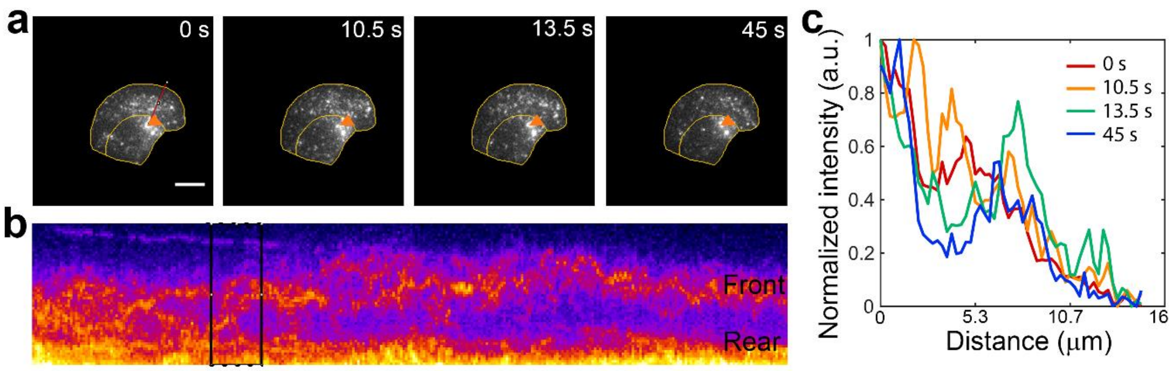

Fig. S10. The QDs rapidly translocate from the rear to the front parts in the lamellipodium. a, Images of QDs in a migrating cell at different time points. The orange triangle points to the region where the QD intensity changes obviously. Scale bar, 10 μm. **b,** Kymograph of the QD intensity along the red line shown in **(a)**. The colors from blue to red represent the increase in intensity. Black box indicates the time period from 10.5 to 13.5 s, during which the translocation of QDs from the rear to front parts in the lamellipodium is completed. **c,** Intensity profile along the red line in (a) is plotted as a function of distance from the rear to the leading edges of lamellipodium. Before 10.5 s, the intensity in the front and rear parts of lamellipodium is relatively equal. Between 10.5 and 13.5 s, the intensity decreases in the rear region while increases in the front region. After 13.5 s, the intensity in the front region is consistently higher than in the rear region, resulting in a pattern with a gap area in the rear part of the lamellipodium.

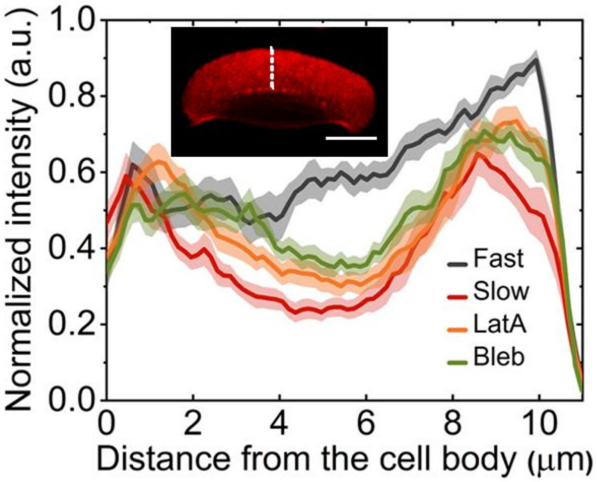

Fig. S11. Intensity profile of actin filaments in lamellipodia labeled by Alexa594-phalloidin. Intensity profiles along a line parallel to cell direction and across the lamellipodium, from the cell body to the leading edge, in persistent migrating cells. Compared with the fast cells (n = 21) in which the actin intensity increases smoothly with the distance, the actin intensities decrease in the rear lamellipodium and then increase in the front in slow (n = 22), LatA-treated (n = 18) and Bleb-treated (n = 18) cells. The actin intensity profiles are consistent with those of cell volume marker CMFDA shown in Fig. 2g. Scale bar, 10 μm. The solid lines and the shaded regions represent mean ± SE.

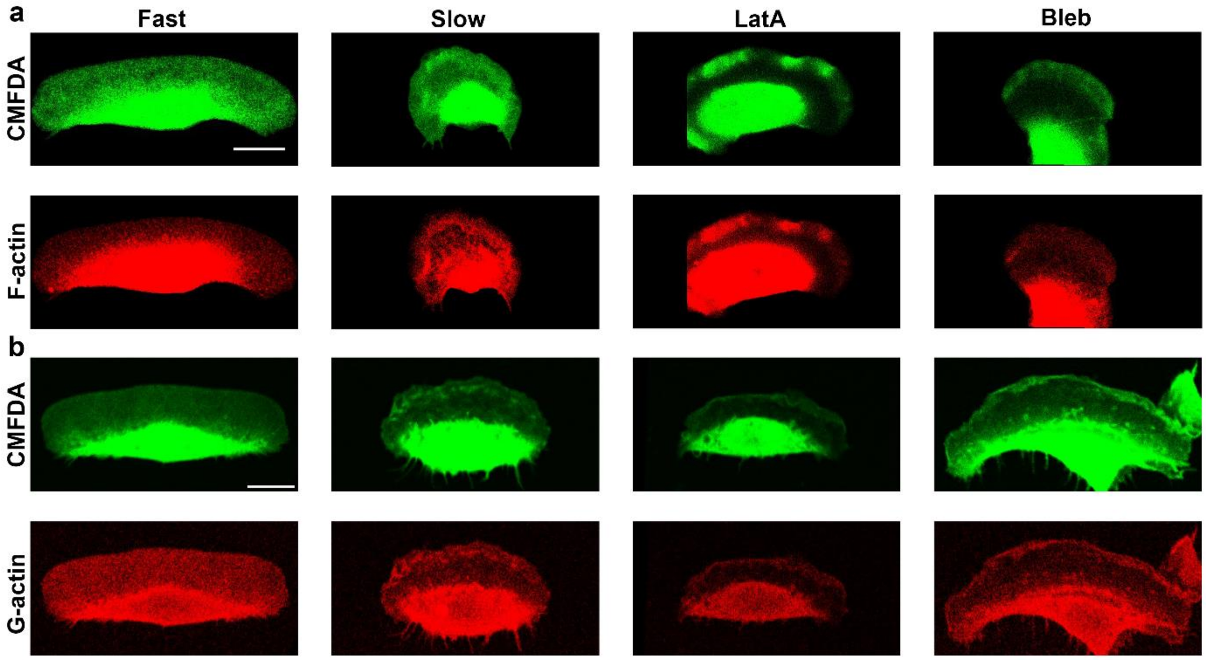

Fig. S12. a, Fluorescence images of CMFDA (upper panels) and F-actin (lower panels) for migrating cells with fast and slow speeds, and for cells treated with 10 nM latrunculin A (LatA), or 50 μM blebbistatin (Bleb). **b,** Fluorescence images of CMFDA (upper panels) and G-actin (lower panels) under the same conditions as those in **(a)**. Scale bar, 10 μm.

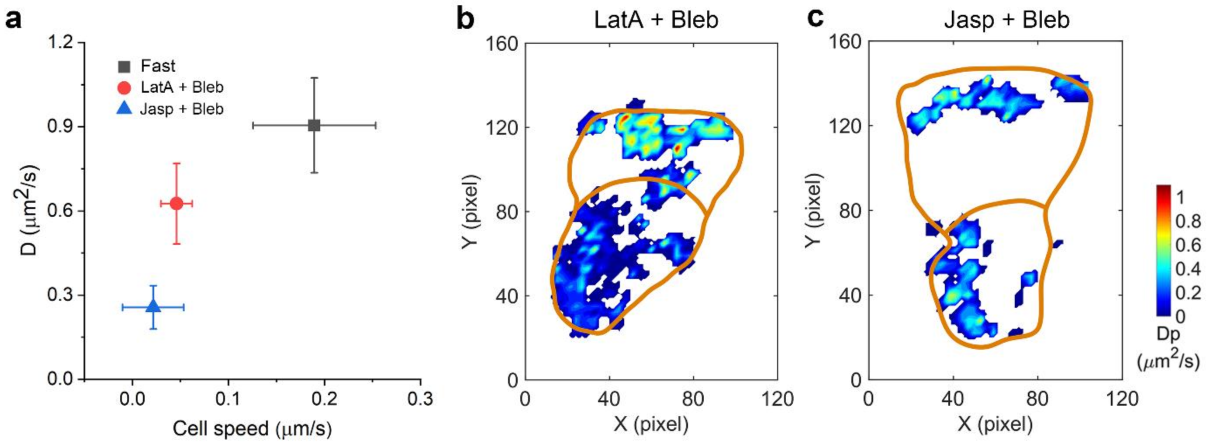

Fig. S13. Similarly reduced intracellular diffusion rates and the diffusion maps with a QD-excluded region are both observed in cells after simultaneous inhibition of actin polymerization and myosin contractility. a, Average diffusion rates and migration speeds for cells with fast speed (n = 6), and for cells treated with 10 nM LatA and 50 μM Bleb (n = 6), or with 1 μM Jasp and 50 μM Bleb (n = 9). **b, c,** Diffusion maps of QDs show the QD-excluded region at the rear lamellipodia of the cells treated with Lat and Bleb **(b)**, or with Jasp and Bleb **(c)**. Data are shown as mean ± SD.

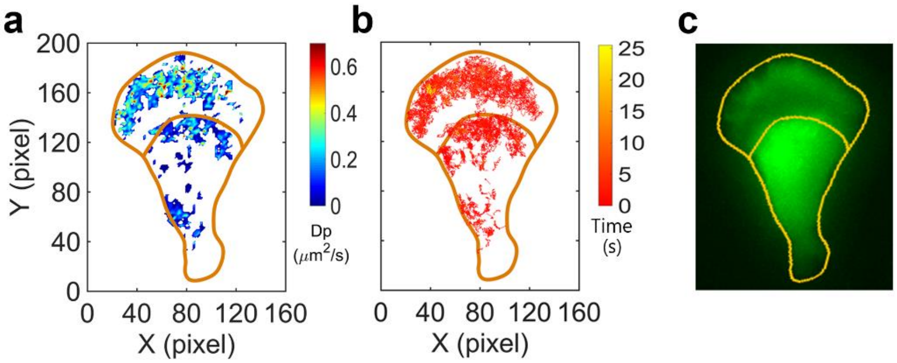

Fig. S14. When a cell is mechanically dragged by other cells, similar excluded regions are observed in the QD intracellular diffusion map **(a)** and the trajectory distribution **(b)**. A gap, which coincides with the excluded region, is found at the rear part of the lamellipodium fluorescence image labeled by CMFDA **(c)**. Note that the cell body is elongated in the *y* direction, due to the connection with other cells below (not included in the figures).

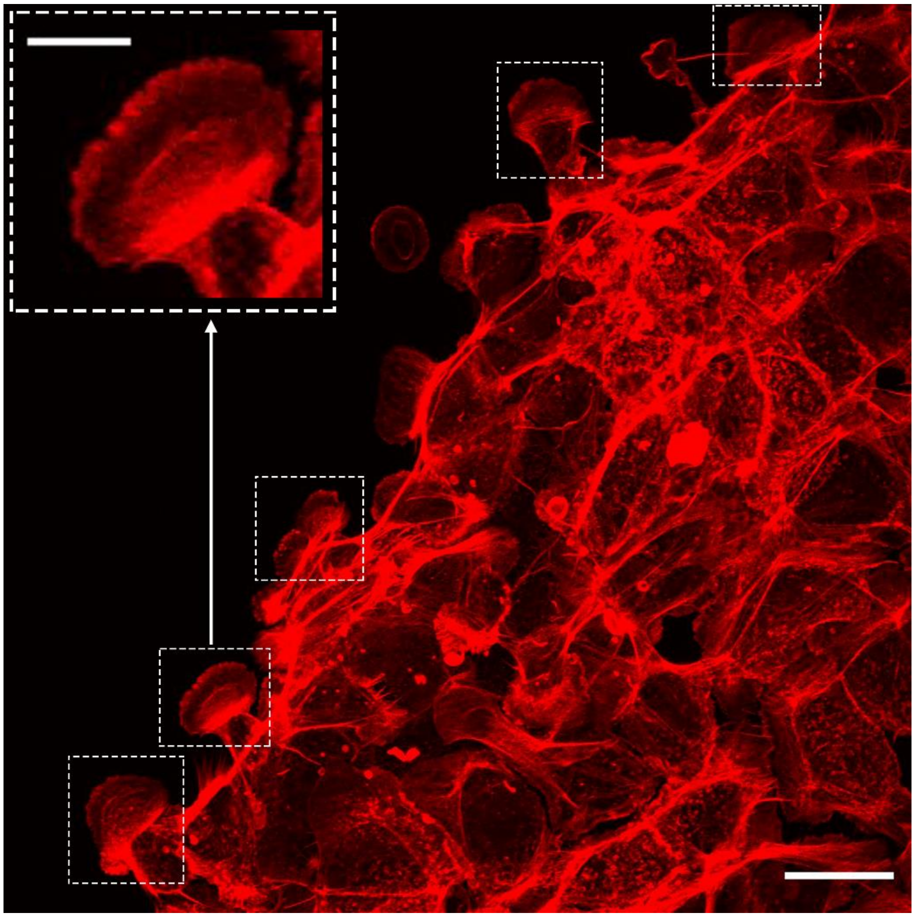

Fig. S15. Fluorescence image of actin filaments in keratocytes. The leading cells (dashed box) at the boundary of the cell monolayer are mechanical dragged by the cells behind. These leading cells all show discontinuous actin distributions in the lamellipodia, with an obvious gap at the rear part near the cell body. Scale bar, 30 μm. Insert, magnified image of a leading cell with the scale bar at 10 μm.

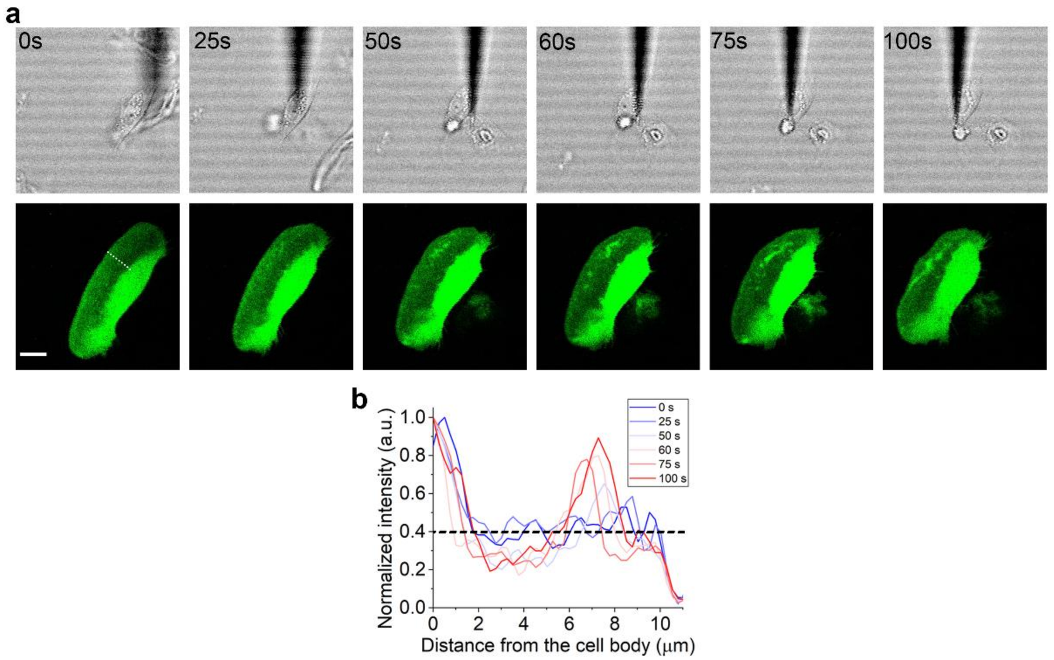

Fig. S16. External mechanical loading slows down the cell migration speed and causes the lamellipodium to change from flat shape to a gap state. A microneedle is used to tether the end of cell body. Serials of bight field and CMFDA fluorescence images for a migrating cell are shown **(a)**, with the intensity profile along the white line from cell body to leading edge determined **(b)**. The intensity is uniform from 2 to 10 μm at the beginning. Upon the loading applied, the intensity for the rear lamellipodium (2 to 6 μm) decreases while the intensity for the front lamellipodium (6 to 10 μm) increases. Scale bar, 10 μm.

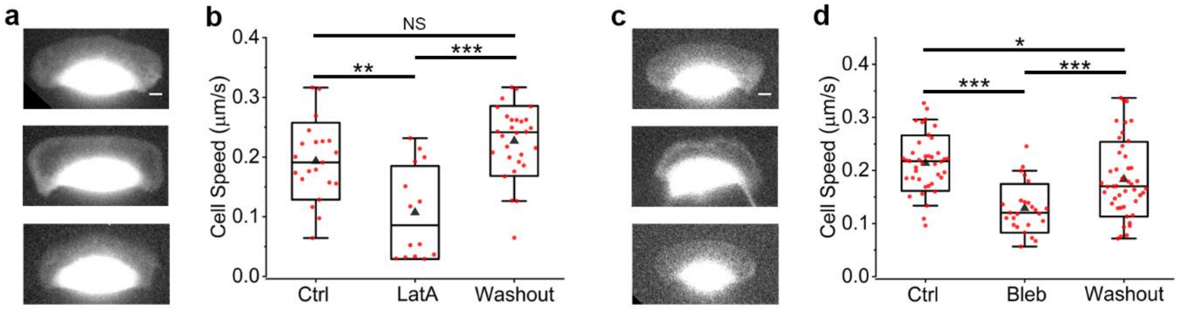

Fig. S17. Lamellipodium returns to the flat shape when the cell speed increases. The live cells are labeled by CMFDA. **a, b,** A representative cell shows a fluorescence gap in the lamellipodium after adding drug latrunculin A (LatA) to inhibit the actin polymerization, and the cell speed decreases accordingly. Then, by washing out the LatA, the gap disappears. Ctrl, $n = 21$; LatA, $n = 14$; Washout, $n = 28$. $p = 0.002$, 5.3E-5, 0.07, for Ctrl and LatA, LatA and Washout, Ctrl and Washout, respectively. **c, d,** Similar to the LatA experiment, the fluorescence gap in lamellipodium is correlated with the cell migration. The cell speed is regulated by using the blebbistatin to attenuate the myosin II activity. Ctrl, $n = 40$; Bleb, $n = 25$; Washout, $n = 45$. $p = 7.0\text{E-}9$, 8.3E-4, 0.03, for Ctrl and LatA, LatA and Washout, Ctrl and Washout, respectively. Scale bar, 10 μm. All the boxes represent SD, where the center lines represent the medians, whiskers represent 1.5×outliers and triangles represent the averages.

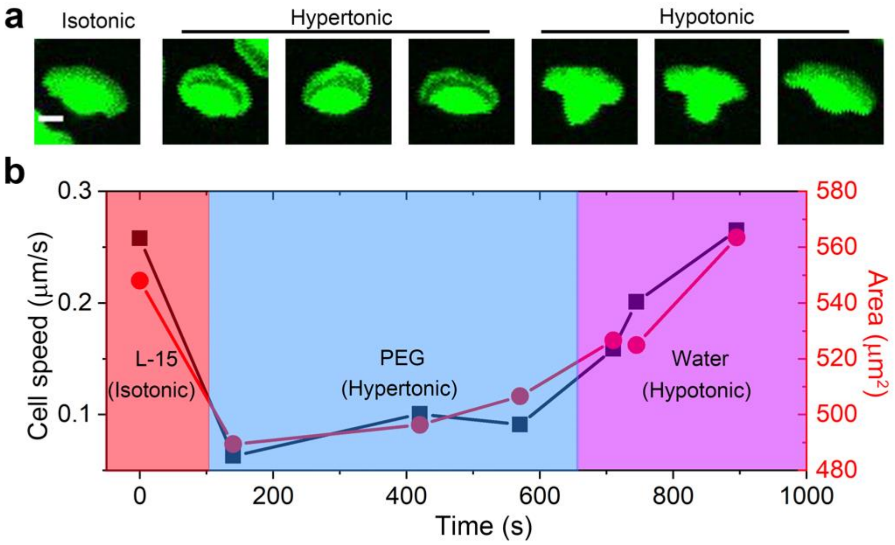

Fig. S18. The cell moving mode is tuned by osmotic pressure of the medium. **a,** CMFDA fluorescent images of the same cell in medium with different osmotic pressures showing the two typical moving modes of keratocytes. The osmotic pressure is changed by adding 1% PEG to the medium or by replacing the medium with double distilled water. **b,** The corresponding speeds and areas of the cell. Scale bar, 10 μm.

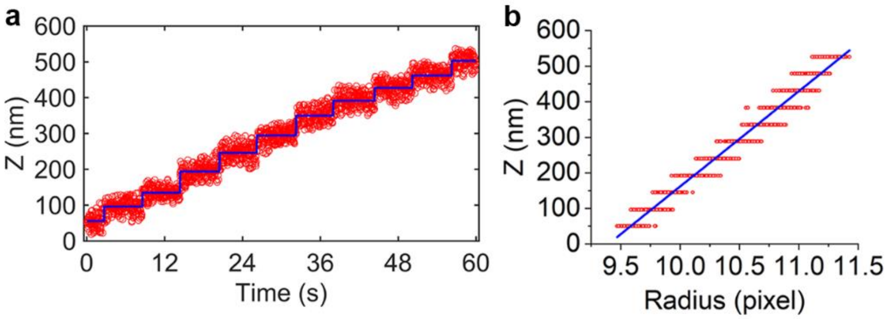

Fig. S19. Calibration between the diffraction ring radius and axial positions for 3D single-particle tracking. a, Single QDs immobilized on coverglass were imaged when the objective was axially shifted at 50-nm steps by a piezoelectric stage. **b,** Linear fit of the *z* coordinate with the corresponding ring radius (r). The obtained calibration is $z = -2519 + 268r$.

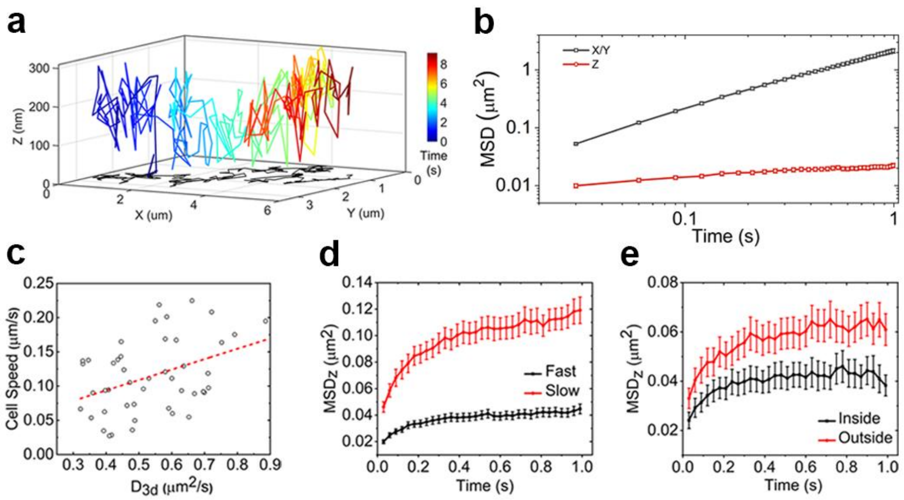

Fig. S20. 3D SPT tracking of QDs in cell lamellipodia. a, Representative 3D trajectories of diffusing QDs. **b,** Comparison of MSD curves for QDs between the *x*/*y* and *z* directions demonstrate the quasi-two-dimensional diffusion in lamellipodia. **c,** Positive correlation between the 3D diffusion rate and the cell speed (cell, n = 46). **d,** Comparison of MSD of diffusing QDs in the *z* direction between fast (n = 15) and slow cells (n = 17). **e,** Comparison of MSD of diffusing QDs in the *z* direction between the inside and outside lamellipodia in turning cells (n = 10). Error bars indicate the SEM.

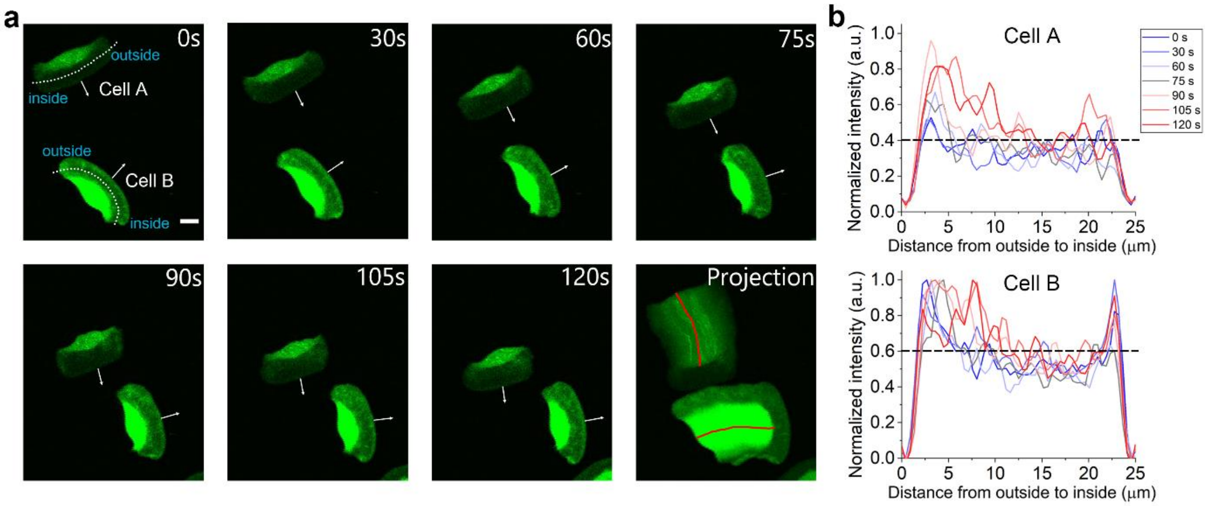

Fig. S21. Turning cells manifest a left-right symmetry breaking in lamellipodium thickness. a, Cell A migrates downward, whereas Cell B migrates from left to right. Their temporal directions at every 30-s interval are indicated by the arrows, which illustrates the cell turning behaviors. **b,** The intensity profiles along the white line from outside to inside parts, in Cell A and B. The lamellipodia are stained by CMFDA to indicate the thickness. For each cell, it is observed that the intensities at both sides of the lamellipodium are similar at the beginning. However, during the turning, the intensity at the outside part (2 to 10 μm) of lamellipodium increases whereas that at the inside part has no obvious change. These results suggest that both cells have left-right symmetry in lamellipodium thickness, while during the turning, the left-right symmetry breaks due to the increase in thickness at the outside lamellipodium. Scale bar, 10 μm.

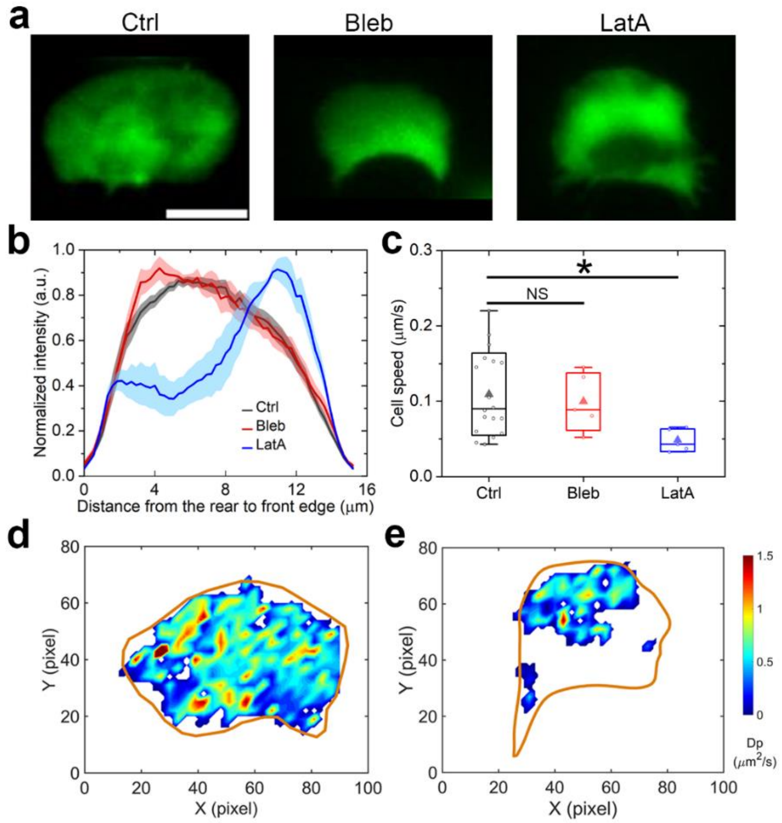

Fig. S22. Lamellipodial fragments show similar changes in both the lamellipodium thickness and intracellular diffusion when their migration speed reduces. a, CMFDA fluorescence images for migrating cells with fast speeds, and for cells treated with 50 μM blebbistatin (Bleb) or 10 nM latrunculin A (LatA). All the fragments are shown to move upwards. Scale bar, 10 μm. **b,** Normalized intensity profiles of CMFDA along a line across the lamellipodium, from the cell body to its leading edge in different conditions (Ctrl, n = 18; Bleb, n = 5; LatA, n = 5). The solid lines and the shaded regions represent mean ± SE. **c,** Speeds of fragments in different conditions (Ctrl, n = 18; Bleb, n = 5; LatA, n = 5). p = 0.72, Ctrl and Bleb; p = 0.024, Ctrl and LatA. All the boxes represent SD, where the center lines represent the medians, whiskers represent 1.5×outliers and triangles represent the averages. Since the loss of myosin contractions, the treatment of Bleb has no obvious effect on the fragment speed and the lamellipodium thickness. **d, e,** Intracellular diffusion maps for 70-kD dextran in fast **(d)** and slow **(e)** fragments.
